# Foliar Essential Oil Glands of *Eucalyptus* Subgenus *Eucalyptus* (Myrtaceae) Are a Rich Source of Flavonoids and Related Non-Volatile Constituents

**DOI:** 10.1371/journal.pone.0151432

**Published:** 2016-03-15

**Authors:** Jason Q. D. Goodger, Samiddhi L. Seneratne, Dean Nicolle, Ian E. Woodrow

**Affiliations:** 1 School of BioSciences, The University of Melbourne, Melbourne, Victoria, Australia; 2 Currency Creek Arboretum, PO Box 808 Melrose Park, Currency Creek, SA, 5039, Australia; Michigan State University, UNITED STATES

## Abstract

The sub-dermal secretory cavities (glands) embedded within the leaves of *Eucalyptus* (Myrtaceae) were once thought to be the exclusive repositories of monoterpene and sesquiterpene oils. Recent research has debunked this theory and shown that abundant non-volatile compounds also occur within foliar glands. In particular, glands of four species in subgenus *Eucalyptus* contain the biologically active flavanone pinocembrin. Pinocembrin shows great promise as a pharmaceutical and is predominantly plant-sourced, so *Eucalyptus* could be a potential commercial source of such compounds. To explore this we quantified and assessed the purity of pinocembrin in glands of 11 species of *E*. subg. *Eucalyptus* using Electro-Spray Ionisation Liquid Chromatography Mass Spectrometry of acetonitrile extracts and Gas Chromatography Mass Spectrometry analyses of hexane extracts of isolated glands which were free from other leaf tissues. Our results showed that the glands of subgenus *Eucalyptus* contain numerous flavanones that are structurally related to pinocembrin and often present in much greater abundance. The maximum concentration of pinocembrin was 2 mg g^-1^ dry leaf found in *E*. *stellulata*, whereas that of dimethylpinocembrin (5,7-dimethoxyflavanone) was 10 mg g^-1^ in *E*. *oreades* and that of pinostrobin (5-hydroxy-7-methoxyflavanone) was 12 mg g^-1^ in *E*. *nitida*. We also found that the flavanones are exclusively located within the foliar glands rather than distributed throughout leaf tissues. The flavanones differ from the non-methylated pinocembrin in the degree and positions of methylation. This finding is particularly important given the attractiveness of methylated flavonoids as pharmaceuticals and therapeutics. Another important finding was that glands of some members of the subgenus also contain flavanone *O*-glucosides and flavanone-β-triketone conjugates. In addition, glands contain free β-triketones, β-triketone heterodimers and chromone *C*-glucosides. Therefore, the foliar glands of this taxonomically distinct group of plants are a rich source of a range of flavonoids and other biologically active compounds with great commercial potential.

## Introduction

One of the most distinctive features of the foliage of *Eucalyptus* (Myrtaceae) trees is the presence of numerous sub-dermal secretory cavities (glands). Until recently, *Eucalyptus* leaf glands were thought to exclusively contain essential oils (a mixture of monoterpenes and sesquiterpenes), but they are now known to house additional non-volatile constituents, only a few of which have been structurally elucidated [1–3]. Remarkably, it has been estimated that the non-volatile compounds in *Eucalyptus* glands can comprise up to 50% of gland lumen volume in some species [4]. In addition, the chemical classes of the non-volatile compounds present in the glands appear to show a level of taxonomic segregation between the five major subgenera of this large genus (>800 species). For example, in a survey of the glandular content of 19 eucalypts from three subgenera, members of the subgenera *Symphyomyrtus* and *Eudesmia* were shown to contain monoterpene acid glucose esters as the most abundant non-volatiles, whereas members of the subgenus *Eucalyptus* were found to contain the flavanone pinocembrin (5,7-dihydroxyflavanone). Interestingly, pinocembrin was not detected at all in *Symphyomyrtus* and *Eudesmia* [3].

This finding that flavonoids occur within the glands of at least some members of subgenus *Eucalyptus* is particularly noteworthy for two reasons. Firstly, research on the biosynthesis, transport and storage of these biologically active compounds will be considerably advantaged by being able to study such processes in isolated, functional glands that are free of other leaf tissues (see [4] for protocol). Secondly, localization of flavonoids within the gland lumen may offer some advantages to rapid selection of high yielding *Eucalyptus* genotypes, which is important given the growing interest in the potential therapeutic and pharmacological applications of the compounds, and a parallel interest in economic production systems (see recent reviews [5–9]). For example, pharmacological studies have shown the flavanone pinocembrin to exhibit properties that may make it amenable for treating cardiovascular diseases and cancer [10]. In addition, pinocembrin has been shown to have the ability to reduce reactive oxygen species and inflammation, and also to have neuroprotective effects such as protecting the brain against damage from ischemic stroke [11]. Furthermore, pinocembrin is readily absorbed following oral administration [12], and *in vitro* studies have shown it to be capable of passing through the blood-brain barrier in a passive transport process [13]. Indeed, the potential therapeutic utility of pinocembrin is so great that in 2008 it was approved by the State Food and Drug Administration of China for clinical trials in patients with ischemic stroke and participants are currently being recruited for phase II clinical trials [14].

At present, flavonoids are largely isolated from plant sources [15]. This is particularly the case for pinocembrin, where it is almost exclusively purified from whole leaf, flower, root or seed extracts from a wide assembly of 16 plant families, but surprisingly in a total of less than 50 species [10]. Pinocembrin can be synthesized chemically [16], but byproducts of excessive hydrogenation are produced during synthesis that may reduce its efficacy [17]. Plant families with the greatest number of pinocembrin-containing species are Myrtaceae with six *Eucalyptus* and one *Syzygium* species, Zingiberaceae with five *Alpinia* species, Piperaceae with three *Piper* species, Verbenaceae with two *Lippia* species and Lauraceae with two *Cryptocarya* species [10]. Within Myrtaceae, the compound has been found in fruit extracts of *Syzygium samarangense* [18], and in addition to the aforementioned foliar gland extracts of *Eucalyptus gregsoniana*, *E*. *muelleriana*, *E*. *pauciflora* and *E*. *olsenii* [3], pinocembrin has also been detected in whole leaf extracts of *E*. *sieberi* [19] and *E*. *fraxinoides* [20]. Furthermore, reanalysis of previously published mass spectral data from whole leaf extracts of a further 11 species of *Eucalyptus* [21] tentatively identified the matching mass of pinocembrin in all species, albeit at low levels in some [22]. Interestingly, all 17 pinocembrin-containing *Eucalyptus* species are members of the subgenus *Eucalyptus*, supporting the notion that members of this subgenus show promise as commercial sources of flavonoids with pharmaceutical and therapeutic potential.

If we are to progress consideration of *Eucalyptus* as possible commercial sources of flavonoids then two initial research questions need to be answered. First, are flavonoids such as pinocembrin largely or exclusively located in the oil glands? Given the ease with which glands can be visualized within intact, fresh leaves [23], an affirmative answer to this question will allow relatively rapid screening for high-yielding plants at both the species and population level, as has been successfully achieved in identifying high-yielding plants for *Eucalyptus* essential oil production [24]. It is noteworthy that in three species from subgenus *Symphyomyrtus*, non-volatile monoterpene acid glucose esters were shown to consistently comprise a constant proportion of gland volume (41, 48 and 49%, respectively) across a range of gland sizes within each species [4]. The second important question involves the purity of individual flavonoids such as pinocembrin: do individuals and species exist in which pinocembrin comprises the entire glandular non-volatile fraction or if not, what types and amounts of other flavonoids co-occur with pinocembrin? Purity at the gland level is clearly an important consideration because it will influence the ease with which pure whole leaf extracts can be obtained. Alternatively, the presence of other flavanones or more broadly, other flavonoids with similar biological activities may also make *Eucalyptus* species attractive targets for commercial harvesting of those compounds.

In this paper we address the issue of flavonoid content and purity within the leaf glands of plants from the subgenus *Eucalyptus*. We selected 11 species of *E*. subg. *Eucalyptus* based on the presence of large and abundant glands in their leaves and the ability to isolate sufficient amounts of these glands for chemical analyses.

## Materials and Methods

### Plant Material

Fully expanded leaves were collected from *Eucalyptus* species growing in Currency Creek arboretum, South Australia (35°25’45”S, 138°45’46”E) in April 2014. Leaves were harvested using a pole pruner and immediately sealed in snap-lock plastic bags and returned to the laboratory on dry ice. Samples were stored at -80°C until analysed.

### Gland isolation and extraction

Foliar glands were isolated from the leaves of collected *Eucalyptus* species following the method described elsewhere [4] using pectinase P-4716 (Sigma-Aldrich, St. Louis, USA) enzyme from *Aspergillus niger*. The enzyme was diluted with distilled water (1:2 v/v). Each leaf was cut into approximately 4 × 20 mm strips and immersed in 1500 μl of diluted pectinase enzyme solution and incubated at 30°C with agitation at 600 rpm for 30 s at 1-minute intervals. The incubation time was altered from 12–24 h for different species depending on the time taken for complete digestion. Incubation was deemed complete when the leaf cuticle with attached epidermal layer could be easily removed with forceps, and mesophyll-free glands separated from vasculature and mesophyll cells via agitation. All glands isolated from leaf strips of each species were collected and two aliquots of 200 glands were randomly selected and immediately ground using vial micropestles, with one dissolved in 200 μl acetonitrile (3 days at 25°C) for extraction of non-volatiles and the other dissolved in 200 μl hexane (3 days at 50°C) for extraction of volatile terpenoids. All extracts were passed through 0.45 μm membrane filters (Phenex PTFE, Phenomenex) to remove any cellular debris.

A separate subset of leaves were scanned for leaf area determinations and leaf mass per unit area calculated by weighing scanned leaves after drying in an oven at 60°C. Gland density was determined as described elsewhere [23]. Average gland lumen volume for each species was estimated by imaging 30 isolated glands and measuring gland diameters using Image J (Version 1.49, NIH, Bethesda, MD, USA). Lumen volumes were then calculated assuming an ellipsoid shape as described elsewhere [4]. The assumption of an average glandular constituent density of 1 μg per nl was made based on the densities of known abundant constituents: 1,8-cineole, 0.92 μg nl^-1^; *p*-cymene, 0.86 μg nl^-1^; β-eudesmol, 0.95 μg nl^-1^; and pinocembrin, 1.39 μg nl^-1^.

To test if the key flavanones were exclusively localized to glands or if they were also present in other leaf tissues, six fully expanded *E*. *nitida* leaves were randomly selected and divided into halves by cutting along the midrib. The area of each leaf half was measured before one half was ground and extracted in acetonitrile, and the other half subjected to enzymatic digestion with only the subsequent isolated glands ground and extracted in acetonitrile. All samples were passed through a 0.45 μm membrane filter to remove any cellular debris. The amount of pinocembrin and pinostrobin per unit leaf area in the leaf and isolated gland extracts were determined using HPLC and statistically compared using paired t-tests (IBM SPSS Statistics version 22, IBM Armonk, NY, USA).

### Chromatographic analyses

Non-volatile extracts of ground glands were fractionated using a Shimadzu Prominence High-Performance Liquid Chromatography (HPLC) system with photo diode array UV detection (190–500 nm). The column used was a Gemini C18 (5 μm, 150 × 4.6 mm; Phenomenex) analytical column eluted at a flow rate of 0.8 ml min^-1^. The eluent system was a gradient of acetonitrile and water (both acidified with acetic acid, 0.1%) from 30–50% acetonitrile over 7 min, followed by 50–95% over 7 min and then isocratic at 95% for a further 12 min, and finally a gradient of 95–100% over 0.25 min. Standards of flavonoids were purchased from Sigma-Aldrich (pinocembrin and pinostrobin; St. Louis, MO, USA) and from Indofine Chemical Company (dimethyl pinocembrin and dimethyl chrysin; Hillsborough, NJ, USA). Standard series were produced for quantification at the following wavelengths: 289 nm (pinocembrin), 288 nm (pinostrobin), 283 nm (dimethylpinostrobin), 263 nm (dimethylchrysin). Alpinetin was quantified at 288 nm using the standard series of pinostrobin.

Electro-Spray Ionisation Liquid Chromatography Mass Spectrometry (ESI-LCMS) of non-volatile extracts was conducted on an Agilent 6520 QTOF MS system (Santa Clara, CA, USA) with a dual spray ESI attached to an Agilent 1200 series HPLC with a diode array detector using the same column and eluent system as above. The MS was operated in both positive and negative modes. Positive mode was operated using the following conditions: nebulizer pressure 37 psi, gas flow-rate 12 l min^-1^, gas temperature 350°C, capillary voltage 4000V, fragmentor 150 and skimmer 65 V. In negative ion mode, a nebulizer pressure of 45 psi and a capillary voltage of 3500 V were used. Mass spectra were collected in the range of 70–1700 *m*/*z*. The analytical method comprised two scan experiments: the first was a full scan with a dwell time of 493 ms spectrum^-1^, followed by a second MS/MS experiment with a dwell time of 493 ms spectrum^-1^ where a collision energy of 20 V was applied. Analytes were also monitored with diode array UV detection at specific wavelengths of 278 nm and 340 nm with the bandwidth set at 4 nm. Chromatograms and mass spectra were evaluated using MassHunter software (Agilent).

Volatiles were extracted from ground glands in hexane containing 100 mg l^-1^ tridecane as an internal standard and incubated at 50°C for 3 days. Samples were then dehydrated with anhydrous Na_2_SO_4_. Hexane samples were analysed by Gas Chromatography with Flame Ionization Detection (GC-FID) using a Perkin Elmer Autosystem XC (Perkin Elmer, Melbourne, Australia) fitted with a Zebron ZB-5 low polarity column (30 m × 0.25 mm i.d., Phenomenex). Helium was used as the carrier gas at a flow rate of 1 ml min^-1^. The injector temperature was 250°C and detector temperature was 220°C. The column temperature was held at 120°C for 1 min following injection, then ramped at 7°C min^-1^ to 180°C and held at that temperature for a further 10 min. GC-FID constituent identification and quantification was based on standard series of key monoterpenes and sesquiterpenes as described elsewhere [25]. β-triketones were quantified based on a standard series of the sesquiterpene caryophyllene oxide (Sigma-Aldrich) due to the unavailability of commercial β-triketone standards.

Gas Chromatography Mass Spectrometry (GC-MS) was also performed on the hexane extracts using an Agilent 7890A GC and 5975C MS (Agilent Technologies, Santa Clara, USA). Samples (1 μl) were injected in splitless mode into a GC-MS system comprised of a Gerstel 2.5.2 autosampler, a 7890A Agilent gas chromatograph and a 5975C Agilent quadrupole MS (Agilent, Santa Clara, USA). The MS was adjusted according to the manufacturer’s recommendations using tris-(perfluorobutyl)-amine (FC-43). The following MS source conditions were used: injection temperature 250°C, transfer line 280°C, ion source 230°C, quadrupole 150°C, 70 eV (EI mode), 2.66 scans s^-1^, and scanning range *m*/*z* 50–600. The GC fractionation was performed on a 30 m VF-5MS column with 0.2 μm film thickness and a 10 m Integra guard column (J & W, Agilent). The carrier gas and temperature program were the same as for the GC-FID. Mass spectra were evaluated using Agilent MSD ChemStation E.02.02.1431 for GC-MS and monoterpenes, sesquiterpenes and β-triketones identified using either the NIST 11 or Adams 2012 mass spectra libraries.

## Results and Discussion

**Fig 1:**
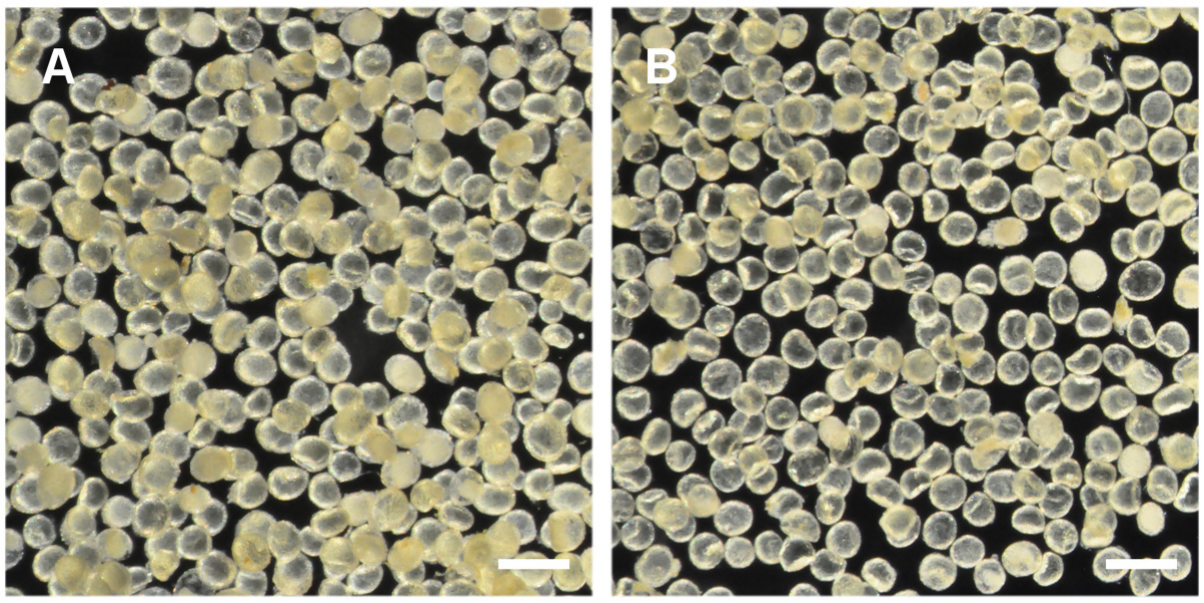
Representative foliar glands isolated from *E*. subg. *Eucalyptus*. A) *E*. *apiculata* and B) *E*. *dendromorpha*. Glands were isolated free from other leaf tissues prior to solvent extraction and chromatographic analyses. Scale bars represent 450 μm.

Glands that were free from other tissues were successfully prepared from 11 *E*. subg. *Eucalyptus* species in this study (Table 1, Fig 1). From each species, 400 glands were randomly collected and half the glands were ground and extracted in acetonitrile and the other half were extracted and ground in hexane. All compounds identified in either solvent were therefore constituents of the gland lumen and/or the secretory cells bounding the lumen, not other leaf tissues.

**Table 1:** Taxonomic classification of the *Eucalyptus* species sampled in this study. Species were chosen based on gland size and abundance and ease of isolation. Average leaf and gland parameters are presented. LMA, leaf mass per unit area.

Species	Subgenus	Section	Series	Subseries	Leaf area (cm^2^)	LMA (g m^-2^)	Gland density (cm^-2^)	Gland volume (μl)
*E*. *oreades*	*Eucalyptus*	*Eucalyptus*	*Fraxinales*		23	319	580	4
*E*. *stellulata*	*Eucalyptus*	*Eucalyptus*	*Longitudinales*		6	206	560	3
*E*. *muelleriana*	*Eucalyptus*	*Eucalyptus*	*Pachyphloiae*		14	273	816	3
*E*. *gregsoniana*	*Eucalyptus*	*Eucalyptus*	*Pauciflorae*		10	275	1400	4
*E*. *falciformis*	*Eucalyptus*	*Eucalyptus*	*Radiatae*		16	239	800	6
*E*. *nitida*	*Eucalyptus*	*Eucalyptus*	*Radiatae*		11	251	830	4
*E*. *approximans*	*Eucalyptus*	Eucalyptus	*Strictae*	*Regulares*	6	208	260	10
*E*. *apiculata*	*Eucalyptus*	Eucalyptus	*Strictae*	*Irregulares*	4	298	530	9
*E*. *dendromorpha*	*Eucalyptus*	Eucalyptus	*Strictae*	*Irregulares*	14	264	576	6
*E*. *suberea*	*Eucalyptus*	*'Frutices'*	*Subereae*		7	182	2100	1
*E*. *brevistylis*	*Eucalyptus*	*Longistylus*	*Pedaria*		14	119	600	2

### Glandular Flavanones

**Fig 2:**
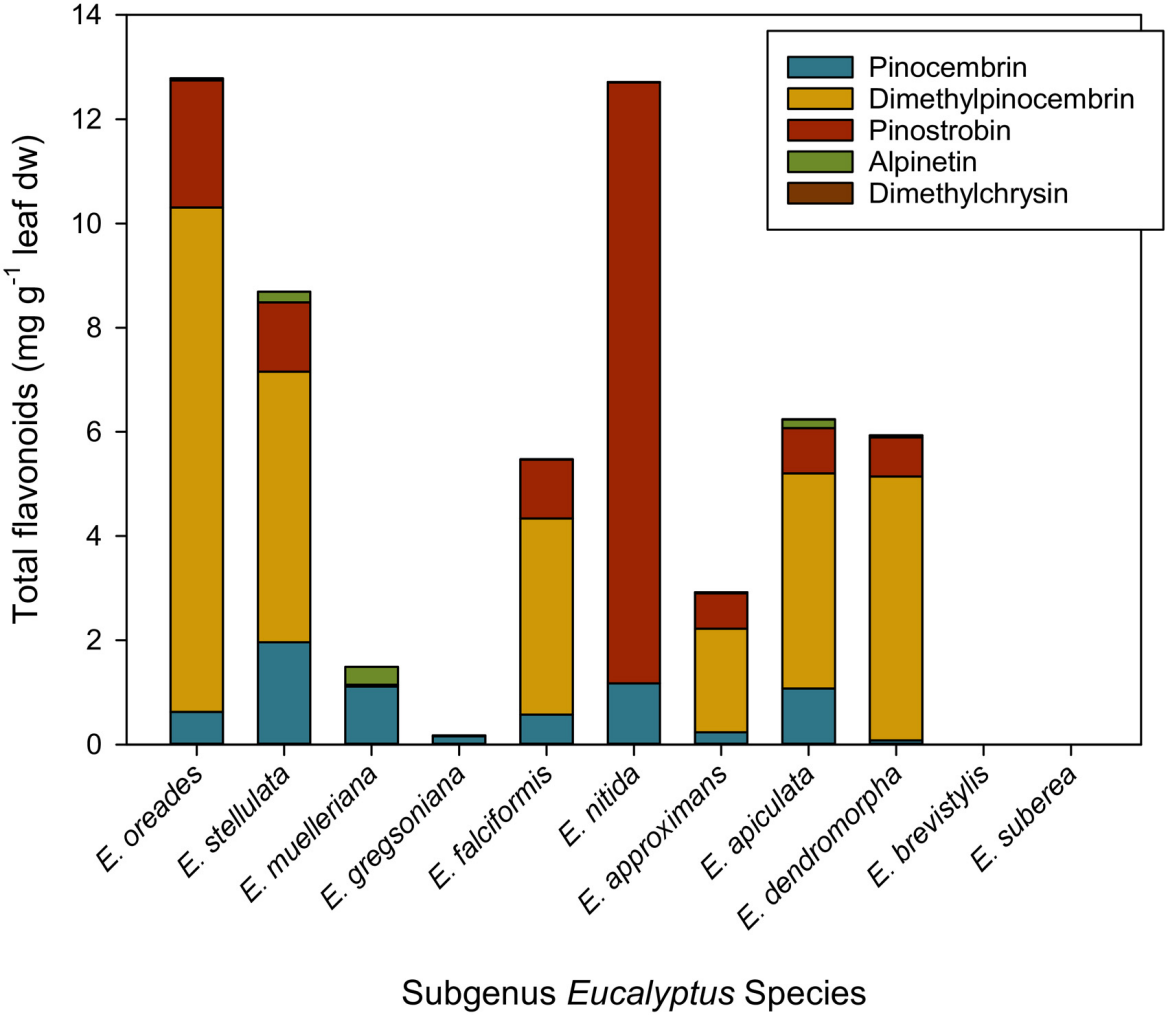
Total quantifiable flavonoids extracted from leaves of *E*. subg. *Eucalyptus* plants. Concentration per unit leaf mass was calculated using flavonoid amount per gland (from HPLC analyses), estimated average gland number per leaf and average leaf dry mass.

From the acetonitrile extracts, we were able to identify and quantify flavonoids based on comparison with a series of commercially available compounds. We found the flavanone pinocembrin to be a key constituent of the non-volatiles in foliar glands of all species from *E*. subg. *Eucalyptus* sect. *Eucalyptus* (see Table 1), a result in agreement with previous analyses of the gland lumen content of four *E*. subg. *Eucalyptus* species [3]. Interestingly, pinocembrin was not the only flavonoid detected in the gland extracts, and it was the most abundant glandular flavonoid in only two species—*E*. *muelleriana* and *E*. *gregsoniana* (Fig 2). We show here for the first time that the related di-methoxylated flavanone dimethylpinocembrin (5,7-dimethoxyflavanone), the structurally similar mono-methoxylated flavanones pinostrobin (5-hydroxy-7-methoxyflavanone) and alpinetin (5-methoxy-7-hydroxyflavanone), and the related dimethoxylated flavone dimethylchrysin (5,7-dimethoxyflavone) are also localized to foliar glands of *E*. sect. *Eucalyptus* species and that some of these compounds are in remarkably high abundance (Fig 2). Interestingly, we were unable to detect any flavonoids in the glands of the two *E*. subg. *Eucalyptus* species examined here that are not part of *E*. sect. *Eucalyptus—E*. *suberea* and *E*. *brevistylis*. Flavonoids have previously been shown to be compartmentalised in analogous plant secretory structures in select plants from other families. In particular, flavones have been detected together with monoterpenes in extracts of glandular trichomes collected from the leaf surface of species in the Lamiaceae (oregano [26] and mint [27]) and the Fabaceae (alfalfa [28]).

The co-occurrence of different methoxylated forms of unsubstituted B-ring flavanones has previously been observed in plants. In particular, pinocembrin, alpinetin, and dimethylpinocembrin were reported in whole leaf extracts of *E*. *sieberi* from *E*. subg. *Eucalyptus* [19]. Similarly, the flavanone dimethylpinocembrin and the flavone dimethylchrysin have been found together in whole leaf extracts of the Myrtaceous shrub *Leptospermum scoparium* [29] and the liverwort *Tylimanthus renifolius* [30], and also in rhizome extracts of *Boesenbergia pandurata* [31].

**Fig 3:**
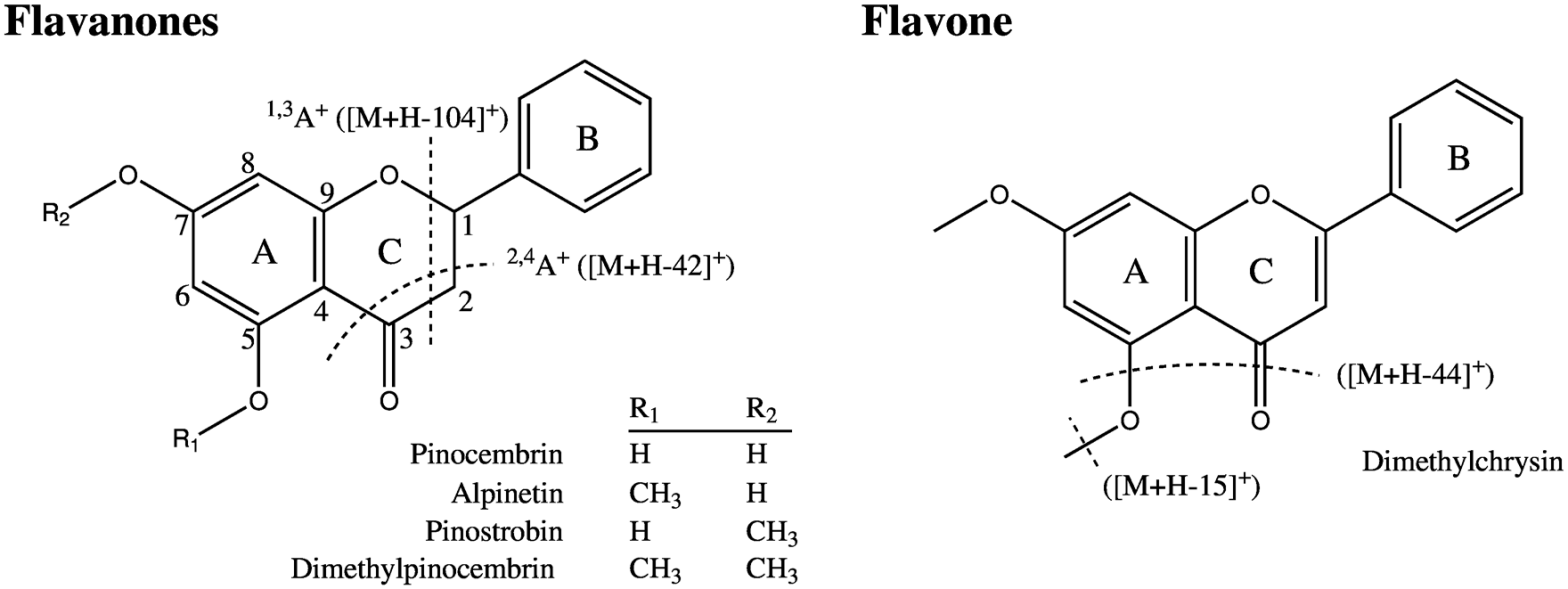
Chemical structures of flavonoids quantified from foliar secretory cavities of 11 species within *E*. subg. *Eucalyptus*. Dashed lines denote characteristic fragmentation patterns of flavanones and flavones observed in ESI-LCMS/MS.

When the amount of flavonoids extracted from glands is presented on a leaf dry mass basis (Fig 3), it is clear that some species contain much higher concentrations of flavonoids in their leaves than others and that the relative proportion of constituent flavonoids also varies. For example, *E*. *nitida* and *E*. *oreades* both contained the highest concentration of total flavonoids (>1.2% w/leaf dw), but *E*. *nitida* contained mostly pinostrobin whereas *E*. *oreades* contained mostly dimethylpinocembrin. Indeed, in six of the species, the latter dimethylated form was the most abundant flavonoid, whereas in all species alpinetin was either the flavanone of lowest abundance or it was not detected (Fig 3).

On a per gland basis *E*. *oreades* glands possessed the most total flavonoids (0.7 μg gland^-1^), followed by *E*. *nitida* and then *E*. *apiculata* (0.4 μg gland^-1^; Table 2). Dimethylpinocembrin was the most abundant flavonoid ranging from 60–84% of total flavonoids in six species (*E*. *oreades*, *E*. *stellulata*, *E*. *falciformis* and the three species from *E*. ser. Strictae—*E*. *approximans*, *E*. *apiculata* and *E*. *dendromorpha*). Pinocembrin was dominant in the two species *E*. *muelleriana* and *E*. *gregsoniana* (76 and 90%, respectively), although it should be noted that total flavanones were very low in both species. Pinostrobin was dominant in *E*. *nitida* (91% of flavanones) and the highest amount of alpinetin was in *E*. *muelleriana* (23%). Interestingly, the two species from *E*. ser. *Radiatae* differed in terms of constituent flavanones with *E*. *falciformis* dominated by dimethylpinocembrin (69%) and *E*. *nitida* dominated by pinostrobin (91%), although both had around 10% pinocembrin.

**Table 2:** Abundant, quantifiable constituents extracted from foliar glands of trees in *E*. subg. *Eucalyptus*. Numbers in parentheses represent percentage abundance of a constituent within each compound class.

Species	Flavanones		Monoterpenes		Sesquiterpenes		β-triketones		Totalμg per gland
	Abundant constituents	μg per gland	Abundant constituents	μg per gland	Abundant constituents	μg per gland	Abundant constituents	μg per gland	
*E*. *oreades*	pinocembrin dimethylether (75%), pinostrobin (19%), pinocembrin (5%)	0.71	*p*-cymene (41%), *trans*-*p*-menth-2-en-1-ol (22%), *trans*-piperitol (16%)	1.01	β-eudesmol (40%), C_15_H_26_O (20%), spathulenol (6%)	0.07	*Not detected*	0.00	1.79
*E*. *stellulata*	pinocembrin dimethylether (60%), pinocembrin (23%), pinostrobin (15%)	0.32	*p*-cymene (61%), 1,8-cineole (21%), d-terpinene (5%)	0.19	spathulenol (23%), viridiflorol (13%), α-caryophyllene (10%)	0.03	*Not detected*	0.00	0.54
*E*. *muelleriana*	pinocembrin (76%), alpinetin (23%), pinocembrin dimethylether (2%)	0.05	α-pinene (79%), 1,8-cineole (11%), *trans*-*p*-menth-2-en-1-ol (3%)	0.24	α-caryophyllene (15%), caryophyllene oxide (11%), spathulenol (10%)	0.04	*Not detected*	0.00	0.33
*E*. *gregsoniana*	pinocembrin (90%), pinocembrin dimethylether (9%), alpinetin (1%)	<0.01	α-pinene (59%), 1,8-cineole (35%), limonene (3%)	0.10	β-eudesmol (50%), C_15_H_26_O (23%), γ-eudesmol (12%)	0.14	*Not detected*	0.00	0.25
*E*. *falciformis*	pinocembrin dimethylether (69%), pinostrobin (21%), pinocembrin (10%)	0.16	1,8-cineole (83%), limonene (6%), α-pinene (4%)	1.27	β-eudesmol (24%), calarene (15%), d-cadinene (7%)	0.03	*Not detected*	0.00	1.46
*E*. *nitida*	pinostrobin (91%), pinocembrin (9%)	0.38	*p*-cymene (44%), 1,8-cineole (20%), piperitone (17%)	0.99	β-eudesmol (19%), spathulenol (18%), C_15_H_26_O (18%)	0.07	*Not detected*	0.00	1.44
*E*. *approximans*	pinocembrin dimethylether (67%), pinostrobin (23%), pinocembrin (8%)	0.24	piperitone (70%),limonene (18%), *trans*-*p*-menth-2-en-1-ol (4%)	1.05	α-gurjunene (23%), aromadendrene (22%), C_15_H_26_O (17%)	0.02	*Not detected*	0.00	1.31
*E*. *apiculata*	pinocembrin dimethylether (64%), pinocembrin (17%), pinostrobin (14%)	0.36	1,8-cineole (45%), *cis*-piperitol (14%), *trans*-*p*-menth-2-en-1-ol (10%)	0.83	caryophyllene oxide (30%), aromadendrene (17%), ledene (10%)	0.02	*Not detected*	0.00	1.21
*E*. *dendromorpha*	pinocembrin dimethylether (84%), pinostrobin (12%), dimethylchrysin (2%)	0.28	*p*-cymene (36%), piperitone (35%), *trans*-*p*-menth-2-en-1-ol (11%)	0.72	trans-caryophyllene (25%), C_15_H_24_ (16%), calarene (13%)	0.03	*Not detected*	0.00	1.03
*E*. *suberea*	*Not detected*	0.00	*trans*-piperitone (trace), cryptone (trace)	<0.01	bicyclogermacrene (48%),C_15_H_24_ (28%)	0.01	conglomerone (60%), agglomerone (30%), isobaeckeol ME (5%)	0.06	0.07
*E*. *brevistylis*	*Not detected*	0.00	*p*-cymene (41%), *trans*-*p*-menth-2-en-1-ol (22%), *trans*-piperitol (16%)	0.01	β-eudesmol (40%), C_15_H_26_O (20%), spathulenol (6%)	0.08	conglomerone (100%)	0.02	0.11

Despite pinocembrin not being the dominant flavonoid in the glands of the majority of species sampled in this study, the maximum concentration of 2 mg g^-1^ leaf dw detected in *E*. *stellulata* remains comparable with commercial plant sources. For example, the concentration of pinocembrin extracted from seeds of the Chinese medicinal plant *Alpinia katsumadai* ranges from as low as 0.02 mg g^-1^ [32] to 2.5 mg g^-1^ [33], and Indian *Curcuma ecalcarata* rhizomes contain up to 4 mg g^-1^ pinocembrin [34]. The pinocembrin abundances described here are also similar to those found recently in whole leaf extracts of the *E*. subg. *Eucalyptus* species *E*. *fraxinoides* and *E*. *sieberi* which contained 2.4 and 3.3 mg g^-1^ pinocembrin, respectively [20]. Interestingly, in the studies on *A*. *katsumadai*, the monomethylated flavanone alpinetin was found in equal [33] or much greater amounts [32] than pinocembrin. This was not the case in any of the *Eucalyptus* species studied here, with only very low levels of alpinetin detected, but it is reminiscent of the high levels of pinostrobin and particularly dimethylpinocembrin we observed to co-occur with pinocembrin. It should be noted that only one tree per species was sampled for this study and given glandular monoterpenes in *Eucalyptus* show quantitative variation in natural populations [35], it is likely that flavanones will show similar intra-specific variability. Future work will aim to identify individuals of species such as *E*. *stellulata* with even greater amounts of pinocembrin and other flavanones in their foliar glands.

A comparison of flavonones extracted from isolated glands of *E*. *nitida* with those extracted from intact leaves (including glands) showed no statistical difference between the concentrations of the abundant flavonoids pinostrobin (t = -0.73, *P* = 0.498) or pinocembrin (t = -1.09, *P* = 0.33). This result suggests that the flavanones are exclusively housed within the glands, rather than distributed throughout different leaf tissues. A similar result was found for the localization of monoterpene acid glucose esters to glands of *Eucalyptus* species in other subgenera [2], and may represent a means of avoiding potential autotoxicity from these biologically active flavonoids.

### Other gland constituents

Comparison of the acetonitrile and hexane extracts enabled quantification of total constituents per gland and the relative proportion of different classes of compounds within the gland lumena. In general, monoterpenes were the most abundant gland constituents in the *E*. subg. *Eucalyptus* species (Table 2). The exceptions to this were *E*. *stellulata*, in which flavanones were the most abundant gland constituents, *E*. *gregsoniana* and *E*. *brevistylis*, in which sesquiterpenes were the most abundant, and *E*. *suberea*, in which β-triketones predominated. As noted above, glands of *E*. *suberea* and *E*. *brevistylis* did not contain detectable flavonoids, but were the only species examined that had β-triketones in their glands. This result suggests that the presence of flavonoids and possibly the absence of free β-triketones in foliar glands may be characteristic of *E*. sect. *Eucalyptus* species. Glands of *E*. *falciformis* possessed the most monoterpenes (1.3 μg gland^-1^), followed by *E*. *approximans*, *E*. *oreades* and *E nitida* (1.0 μg gland^-1^). Overall, *p*-cymene, α-pinene and 1,8-cineole were the most abundant monoterpene constituents found in glands. In general, glands of all species contained low levels of sesquiterpenes, the highest being *E*. *gregsoniana* (0.1 μg gland^-1^). The major sesquiterpene observed was β-eudesmol ranging from 19–50% of total sesquiterpenes.

**Fig 4:**
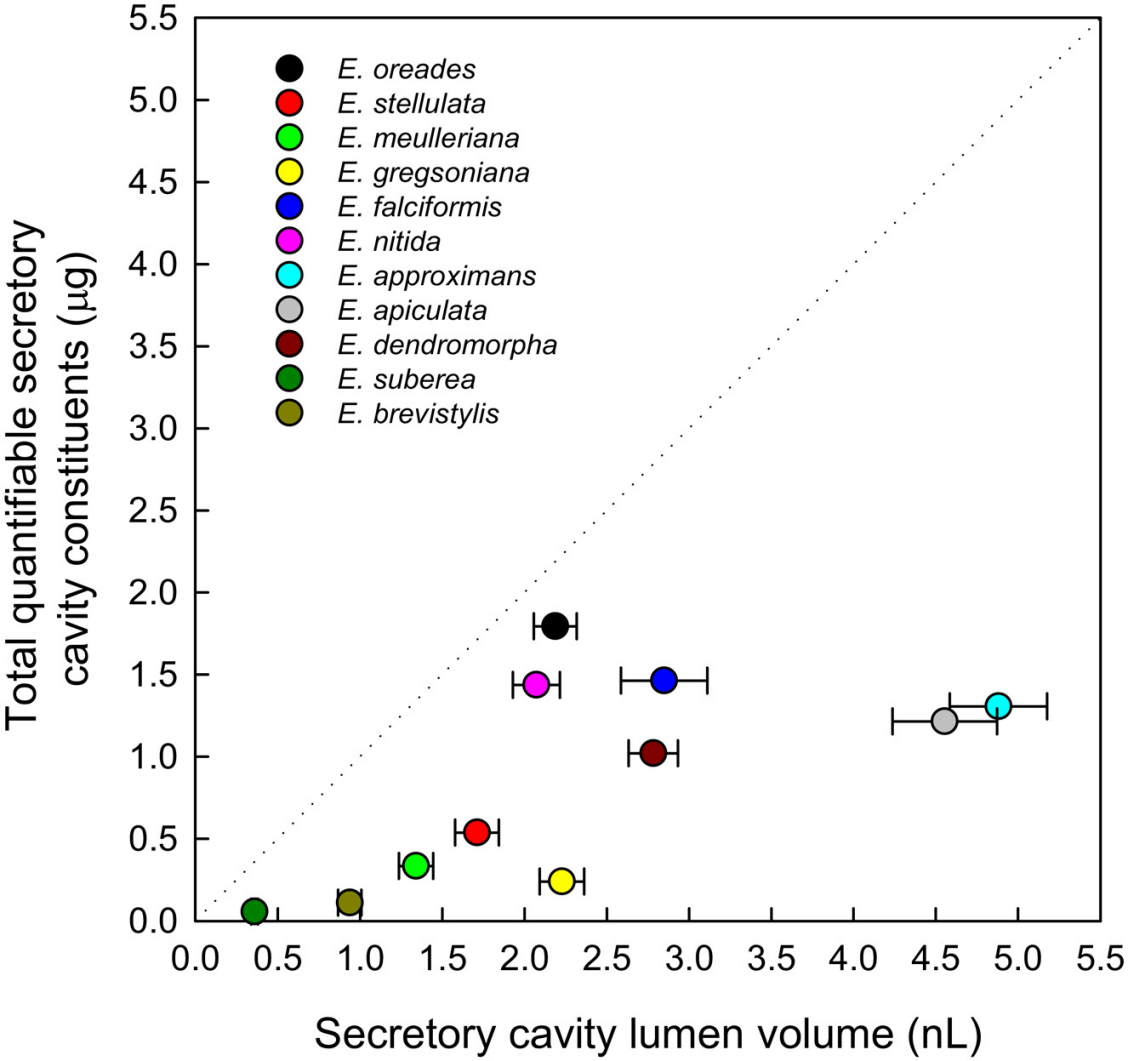
Relationship between quantifiable constituents per gland and the estimated gland lumen volume for 11 species within *E*. subg. *Eucalyptus*. Quantifiable constituents were flavanonoids, monoterpenes, sesquiterpenes and triketones. Lumen data are the means (± 1 SE) of 30 cavities. Dotted line represents an idealised 1 to 1 relationship between lumen volume and total gland constituents, assuming an average constituent density of 1 μg per nl.

The sum of all quantifiable constituents per gland (Table 2) was plotted against the average gland lumen volume for each species (Fig 4). In some species, the total quantifiable constituents accounted for most of the gland lumen volume (assuming an average constituent density of 1 μg per nl), but in all species there was a portion of volume unaccounted for, and indeed in species such as *E*. *gregsoniana*, *E*. *approximans* and *E*. *apiculata*, the known constituents accounted for only a low proportion of gland lumen volume. This suggests that glands of the species studied contained other non-volatile compounds. This notion was reinforced by the number of unknown PDA peaks observed with HPLC fractionation of the acetonitrile extracts.

**Fig 5:**
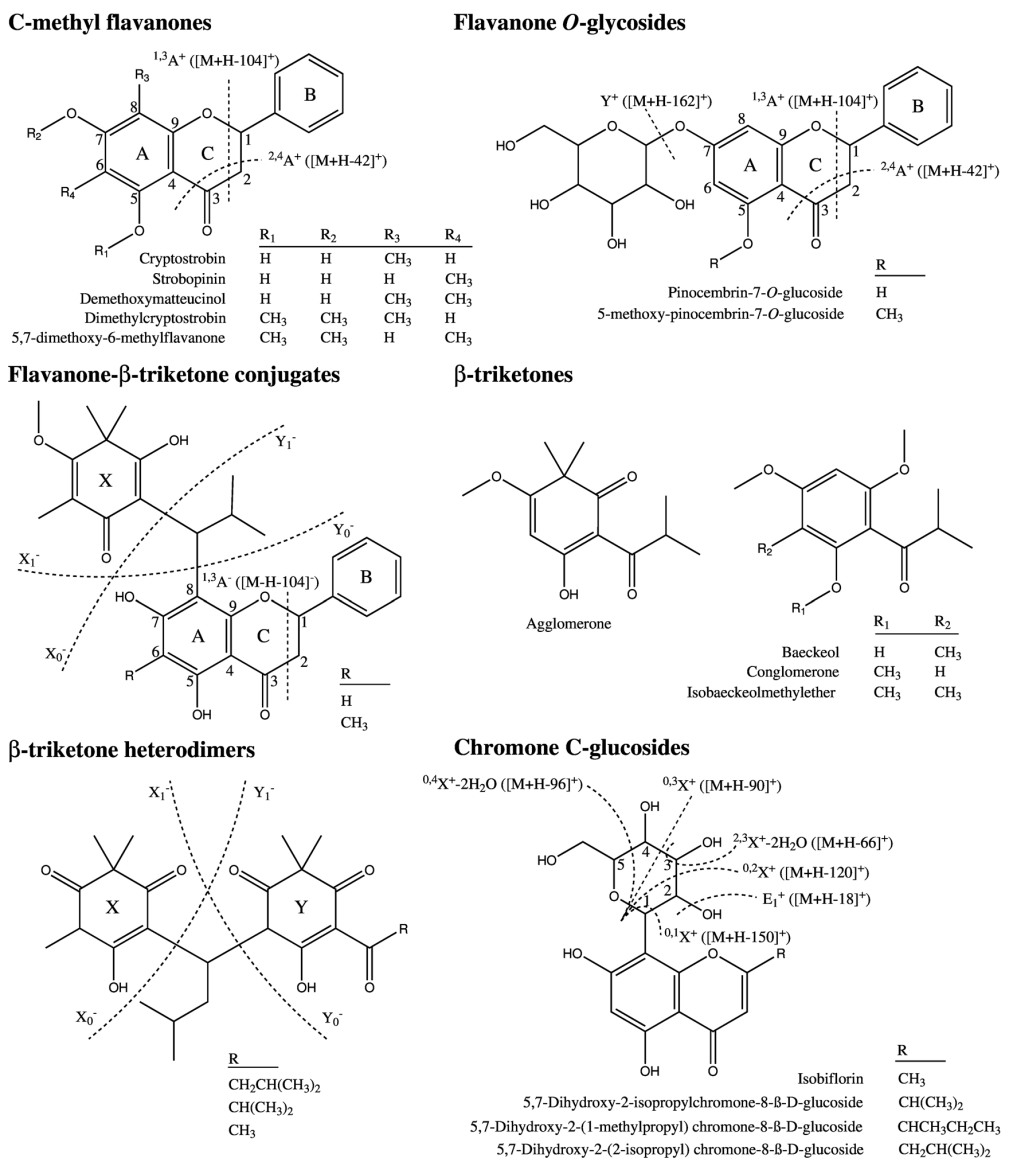
Compounds identified from extracts of isolated glands of *E*. subg. *Eucalyptus*. Dashed lines denote characteristic fragmentation patterns observed in ESI-LCMS/MS. Identifications are based on known compounds from genus *Eucalyptus* or family Myrtaceae.

We used ESI-LCMS/MS to identify unknown constituents in glands, focusing on the characteristic fragmentation patterns of particular compound classes and structures known to occur in *Eucalyptus* or more broadly within Myrtaceae. Firstly, unsubstituted B-ring flavanones characteristically produce fragments with neutral losses of 104 and 42 Da via heterocyclic C-ring fragmentation (Fig 2; [36, 37]). Searching for compounds with these characteristic losses in the mass spectra of the acetonitrile extracts showed many putative flavanones and more complex compounds containing flavanone moieties. In particular, a number of C-methyl flavanones were observed (Fig 5, S1 Fig), and this class of compound was particularly abundant in the *E*. *muelleriana* gland extract. Many of these compounds have previously been detected in the family Myrtaceae. For example: (1) strobopinin (5,7,dihydroxy-6-methylflavanone) has been found in *Corymbia torelliana* fruit resins [38]; (2) cryptostrobin (5,7,dihydroxy-8-methylflavanone), strobopinin, dimethylstrobopinin (5,7,dimethoxy-6-methylflavanone), and demethoxymatteucinol (6,8-dimethylpinocembrin) together with pinostrobin and pinocembrin have been found in extracts of whole *Campomanesia adamantium* leaves [39]; (3) strobopinin and desmethoxymatteucinol have been found in *Heteropyxis canescens* leaves [40]; (4) strobopinin, cryptstrobin, demethoxymattuecinol and 7-hydroxy-5-methoxy-6,8-dimethylflavanone together with pinocembrin have been found in *Sygium samarangense* leaves [41]; and (5) strobopinin together with dimethylpinocembrin have been found in *Leptospermum scoparium* leaves and stems [42]. It is particularly noteworthy that recent work on *L*. *scoparium* and *L*. *morrisonii* used Raman spectroscopy on intact leaves to show that both the β-triketone grandiflorone and unidentified *C*-methyl flavanones were compartmentalized within embedded foliar glands [43].

Further analysis of glandular extracts using LC-MS/MS showed the presence of larger, more complex compounds that contained flavanone moieties. For example, *E*. *stellulata* glands contained two flavanone-glucosides. These compounds were identified based on the characteristic fragmentation of unsubstituted B-ring flavanones, together with the characteristic neutral loss of glucose (162 Da) from glucosides [44]. The first compound eluted at 6.4 min with a *m*/*z* of 419.13 [M + H]^+^ and 417.13 [M − H]^−^ in the respective ionization modes, corresponding to a molecular formula of C_21_H_22_O_9_. The loss of glucose from this compound via MS_2_ fragmentation produced a flavanone moiety with *m*/*z* 257.08 [M + H − 162]^+^ and 255.07 [M − H − 162]^−^ corresponding to pinocembrin, which then underwent subsequent fragmentation resulting in neutral losses of 104 Da in both ionization modes (S2 Fig). The second compound was of lower abundance based on PDA and ion current and eluted at 8.6 min with a *m*/*z* of 433.10 [M + H]^+^ corresponding to a molecular formula of C_22_H_24_O_9_. This compound fragmented with the loss of glucose to pinostrobin (*m*/*z* 271.09 [M + H − 162]^+^) which further fragmented with a neutral loss of 104 Da to *m*/*z* 167.04 [M + H − 162–104]^+^. The likely structures of these compounds are pinocembrin-7-*O*-glucoside and 5-methoxy-pinocembrin-7-*O*-glucoside (Fig 5), which have been shown to co-occur in aerial parts of *Penthorum chinense* (Penthoraceae; [45]). Moreover, both compounds have been found individually in many different plant families, but not previously in Myrtaceae. Interestingly, neither of these flavanone-glucosides was detected in any of the other *Eucalyptus* species in this study. It is noteworthy that flavonoid glucosides have previously been identified from extracts of monoterpene producing glandular trichomes of *Phillyrea latifolia* (Oleaceae; [46]).

LC-MS/MS analysis of the acetonitrile extract from *E*. *meulleriana* showed its glands to contain a suite of other compounds containing flavanone moieties. The parent masses and MS_2_ fragments of these compounds strongly suggest they are flavanone-triketone conjugates linked by an *iso*-butyl moiety. In particular, abundant compounds were detected at 16.9 min, with a *m*/*z* of 491.21 [M − H]^−^ corresponding to C_29_H_32_O_7_ and fragmenting to *m*/*z* [M − H]^−^ 181.09, 309.11 and 255.07 (pinocembrin; S3A Fig), and at 18.0 min, with a *m*/*z* of 505.23 [M − H]^−^ corresponding to C_30_H_34_O_7_ and fragmenting to *m*/*z* [M − H]^−^ 181.09, 323.13 and 269.08 (pinostrobin; S3B Fig). There is a consistent difference of 55 Da between the fragment ion *m*/*z* 181 and the loss of 236 Da (*m*/*z* 255 fragment) from the first compound, and the fragment ion *m*/*z* 181 and the loss of 236 Da (*m*/*z* 269 fragment) from the second compound suggesting fragmentation occurs either side of an *iso*-butyl bridge in both compounds (as shown in the fragmentation scheme in Fig 5, S3 Fig). A compound matching the parent mass and fragmentation pattern of compound 2 is known from the myrtaceous species *Baeckia frutescens* [47].

Similar flavanone-triketone conjugates are known from the literature, but none perfectly matched the mass spectra of the other compounds detected in *E*. *muelleriana*. For example, structurally similar compounds have been isolated from extracts of aerial parts of the myrtaceous shrubs *Kunzea ambigua* and *K*. *baxterii* in which the flavanone and β-triketone moieties are linked by an *iso*-pentyl, rather than *iso*-butyl moiety [48]. Kunzeanones A and B have also been isolated from leaves of *K*. *ambigua* and have flavanone and β-triketone moieties linked via two bridges: an *iso*-pentyl moiety and an oxygen bridge through the double bonded oxygen of the β-triketone and 7-hydroxyl of flavanone [49]. It should be noted that these single and double bridge compounds may be tautomers. Similarly, leaves from the myrtaceous shrub *Luma chequen* contain lumaflavanones A-C, structures with a flavanone bonded to a β-triketone through both an *iso*-propyl bridge and an oxygen bridge [50]. Finally, leucadenones A-D extracted from leaves of *Melaleuca leucadendron* have a β-triketone and a flavanone linked by a phenyl moiety [51]. Despite none of these structures being detected in either *E*. *muelleriana* or the other species examined here, the mass spectral data and fragmentation patterns suggest many similar compounds are likely to be present in the glands of *E*. subg. *Eucalyptus* trees and may be present in relatively high abundances.

Non-flavonoid glandular constituents were also detected in *E*. subg. *Eucalyptus* based on characteristic mass spectral fragmentation patterns. In addition to the free β-triketones detected in the hexane extracts of *E*. *brevistylis* and *E*. *suberea* glands, and the flavanone-triketone conjugates of *E*. *muelleriana* glands, the acetonitrile extract of *E*. *gregsoniana* also contained β-triketone heterodimers linked by an *iso*-pentyl moiety (Fig 5). These include compounds with high abundance (according to absorbance at 278 nm) detected at 16.8 min with a *m*/*z* of 431.21 corresponding to C_24_H_32_O_7_, 17.6 min with a *m*/*z* of 445.22 [M − H]^−^ corresponding to C_25_H_34_O_7_, 18.5 min with a *m*/*z* of 459.24 [M − H]^−^ corresponding to C_26_H_36_O_7_, and at 21.3 min with a *m*/*z* of 473.25 [M − H]^−^ corresponding to C_27_H_38_O_7_. These compounds are likely semimyrtucommulones identified from the Myrtaceous shrubs *Myrtus communis* [52, 53] and *Kunzea sinclairii* and *K*. *ericoides* [54] in which the syncarpic acid and phloroglucinol moieties fragment characteristically on either side of the *iso*-pentyl bridge (Fig 5; S4 Fig). A number of unknown compounds with a similar fragmentation pattern were observed in *E*. *gregsoniana* and other *E*. subg. *Eucalyptus* species, suggesting additional β-triketone heterodimers likely occur in the glands of the subgenus.

A final group of non-flavonoid glandular constituents exhibited the highly characteristic mass spectral fragmentation pattern of *C*-glucosides. In contrast to the abundant neutral loss of glucose (162 Da) commonly observed with the cleavage of the *O*-link in *O*-glucosides, ESI-MS/MS fragmentation of *C*-glucosides is restricted almost exclusively to the glycan resulting in fragments with characteristic neutral losses of 18, 66, 90, 96, 120 and 150 Da, which may be associated with additional water losses to give further neutral losses of 36, 54 and 138 Da (Fig 5, S5 Fig; [55]). Moreover, the relative abundance of fragments is diagnostic of the presence of 6-*C* or 8-*C*-glucosides. In particular, 6-*C*-glucosides show an increased abundance of 0,2X^+^: [M + H − 120]^+^ relative to 0,1X^+^: [M + H − 150]^+^ (Fig 5, S5 Fig; [56]), whereas 8-*C*-glucosides show losses of ≥96 Da without the smaller fragments associated with 6-*C*-glucoside fragmentation [55]. Based on this, and the calculated mass of the aglycone, the *Eucalyptus* glands are highly likely to contain up to four 6-*C* chromone glucosides, differing in the hydrocarbon chains attached to the chromone moiety.

The 6-*C* chromone glucoside isobiflorin was detected at 2.2 min with a *m*/*z* of 355.09 [M + H]^+^ corresponding to C_16_H_18_O_9_ (S5 Fig). The compound was detected in gland extracts of all species examined, albeit at lower levels (based on EIC) in *E*. *approximans*, *E*. *dendromorpha* and *E*. *suberea*. Isobiflorin appears to be relatively common in Myrtaceae and has previously been detected in leaves of *Eucalyptus globulus* [57], *E*. *cypellocarpa* [58], *Baeckea frutescens* [59] and *Eugenia caryophyllata* [60], and in buds of *Syzygium aromaticum* [61] and bark of *S*. *johnsonii* [62].

A related 6-*C* chromone glucoside was detected at 2.4 min with a *m*/*z* of 383.13 [M + H]^+^ corresponding to C_18_H_22_O_9_. This compound was found in at least trace amounts in all species except *E*. *stellulata* and was particularly prevalent (based on EIC) in *E*. *brevistylis*, *E*. *gregsoniana*, and *E*. *muelleriana*. As with isobiflorin, it has previously been observed in a number of myrtaceous species including the bark of *Eucalyptus globulus* [57], *E*. *grandis*, *E*. x *urograndis* and *E*. *maidenii* [63], and leaves of *Baeckea frutescens* [59, 64] and *Kunzea ambigua* [65].

Finally, two 6-*C* chromone glucoside isomers were detected at 2.6 and 4.3 min with *m*/*z* of 397.15 [M + H]^+^ corresponding to C_19_H_24_O_9_. The two isomers were only detected in *E*. *gregsoniana* glandular extracts, but have previously been found individually in *Eucalyptus* with the *C*-glucoside possessing a methlpropyl chain on the chromone moiety found in *E*. *grandis*, *E*. x *urograndis* and *E*. *maidenii* bark [63], and the *C*-glucoside with an isopropyl chain on the chromone found in *E*. *maidenii* branches [66].

## Conclusions

The results presented here show that the foliar oil glands of *Eucalyptus* species from the subgenus *Eucalyptus* contain numerous flavanones that are structurally related to pinocembrin and often present in much greater abundance. We also show that these flavanones are exclusively located within the foliar glands rather than distributed throughout leaf tissues. The flavanones differ from the non-methylated pinocembrin in the degree and positions of methylation of both the A-ring hydroxyls and carbons. This finding is particularly important given the attractiveness of methylated flavonoids as pharmaceuticals and therapeutics due to their improved intestinal absorption [67], enhanced bioactivities [68], and increased metabolic stability [69], relative to non-methylated forms. We show for the first time glands of some *E*. subg. *Eucalyptus* members also contain flavanone *O*-glucosides and flavanone-β-triketone conjugates. In addition, glands contain free β-triketones, β-triketone heterodimers and chromone *C*-glucosides, together with their well characterized complements of monoterpene and sesquiterpene oils. Therefore, the foliar glands of this taxonomically distinct group of plants are a rich source of a range of flavonoids and other biologically active compounds with great commercial potential.

## Supporting Information

S1 Fig Representative mass spectra of putative flavanones and *C*-methyl flavanones detected from *E*. *meulleriana* glands. (PDF)

S2 Fig Representative mass spectra of a putative flavanone *O*-glucoside from *E*. *stellulata* glands. (PDF)

S3 Fig Representative mass spectra of putative flavanone β-triketone conjugates from *E*. *muelleriana* glands. (PDF)

S4 Fig Representative mass spectra of putative β-triketone heterodimers from *E*. *gregsoniana* glands. (PDF)

S5 Fig Representative mass spectra of a putative chromone *C*-glucoside from *E*. *gregsoniana* glands. (PDF)
