# Correction: Foliar Essential Oil Glands of *Eucalyptus* Subgenus *Eucalyptus* (Myrtaceae) Are a Rich Source of Flavonoids and Related Non-Volatile Constituents

**DOI:** 10.1371/journal.pone.0155568

**Published:** 2016-05-09

**Authors:** Jason Q. D. Goodger, Samiddhi L. Senaratne, Dean Nicolle, Ian E. Woodrow

The second author’s last name is spelled incorrectly. The correct name is: Samiddhi L. Senaratne. The correct citation is: Goodger JQD, Senaratne SL, Nicolle D, Woodrow IE (2016) Foliar Essential Oil Glands of *Eucalyptus* Subgenus *Eucalyptus* (Myrtaceae) Are a Rich Source of Flavonoids and Related Non-Volatile Constituents. PLoS ONE 11(3): e0151432. doi:10.1371/journal.pone.0151432
