# Supplementary material for: Foliar Essential Oil Glands of Eucalyptus Subgenus Eucalyptus (Myrtaceae) Are a Rich Source of Flavonoids and Related Non-Volatile Constituents
Source: PLoS One. 2016 Mar 15;11(3):e0151432. doi: 10.1371/journal.pone.0151432 (PMC4792381; doi:10.1371/journal.pone.0151432)
Supplement: S1 Fig — (PDF) [file pone.0151432.s001.pdf]

Supporting Information

S1 Figure. Representative mass spectra of putative flavanones and *C*-methyl flavanones detected in gland extracts of *E. meulleriana*

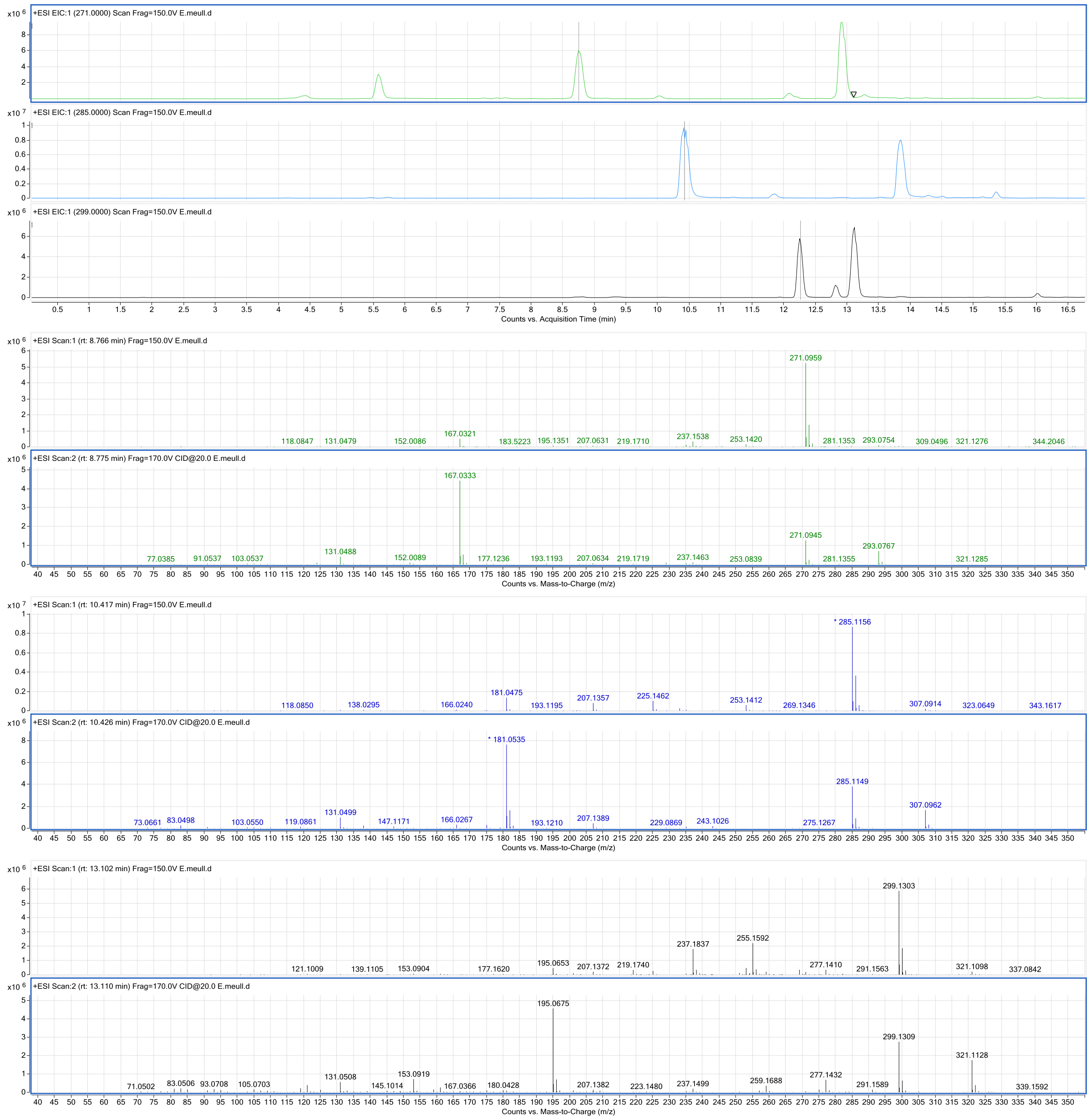

S1 Fig. Multiple flavanones and *C*-methyl flavanones detected in gland extracts of *E. meulleriana* at *m/z* $[M+H]^+$ 271, 285 and 299 using ESI-LCMS/MS in positive mode. All compounds show the characteristic loss of 104 Da of flavanones.
