# Supplementary material for: Foliar Essential Oil Glands of Eucalyptus Subgenus Eucalyptus (Myrtaceae) Are a Rich Source of Flavonoids and Related Non-Volatile Constituents
Source: PLoS One. 2016 Mar 15;11(3):e0151432. doi: 10.1371/journal.pone.0151432 (PMC4792381; doi:10.1371/journal.pone.0151432)
Supplement: S2 Fig — (PDF) [file pone.0151432.s002.pdf]

S2 Figure. Representative mass spectra of a putative flavanone *O*-glucoside from *E. stellulata* glands

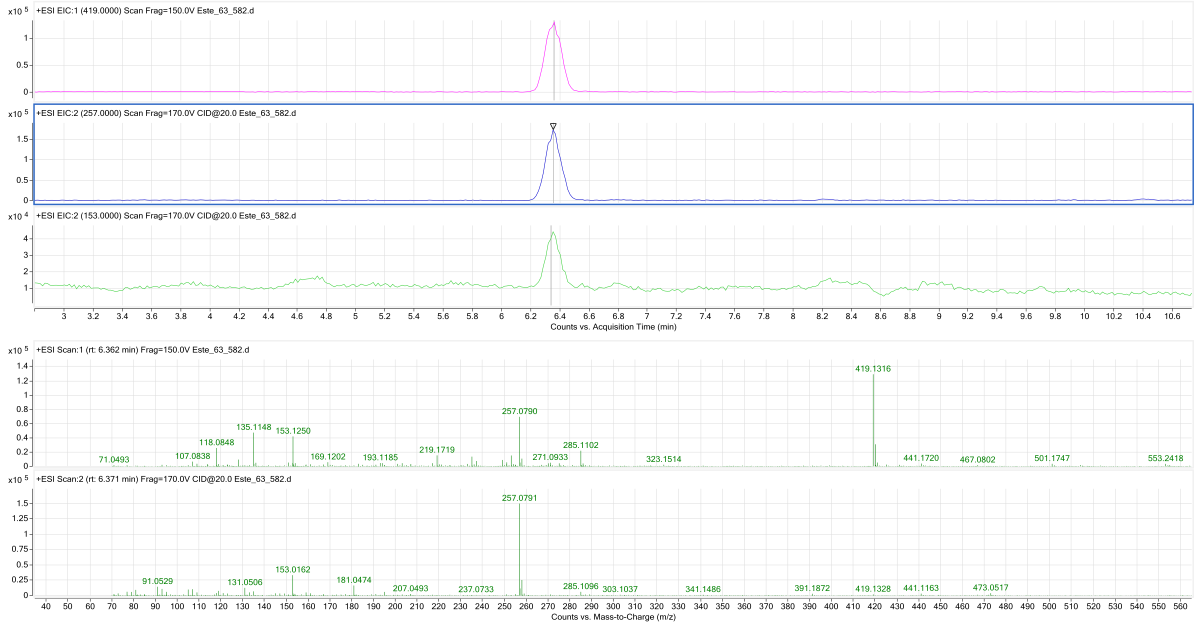

S2A Fig. A flavanone *O*-glucoside from *E. stellulata* glands observed with *m/z* 419 $[M+H]^+$ using ESI-LCMS/MS in positive mode. Fragmentation results in the loss of glucose to yield the flavanone aglycone at *m/z* 257 $[M+H]^+$, which further fragments with a characteristic loss of 104 Da to *m/z* 153.

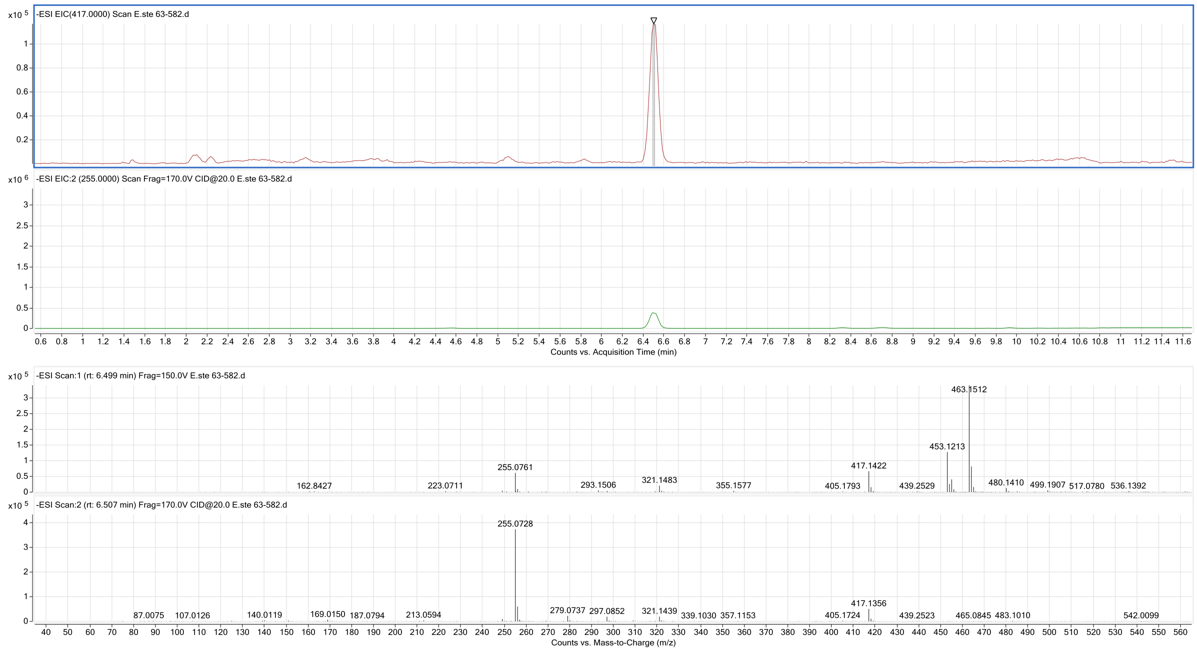

S2B Fig. The same flavanone *O*-glucoside from *E. stellulata* glands observed with *m/z* 417 $[M-H]^-$ using negative mode. Fragmentation results in the loss of glucose (162 Da) yielding the flavanone aglycone with *m/z* 255 $[M-H]^-$.
