# Supplementary material for: Foliar Essential Oil Glands of Eucalyptus Subgenus Eucalyptus (Myrtaceae) Are a Rich Source of Flavonoids and Related Non-Volatile Constituents
Source: PLoS One. 2016 Mar 15;11(3):e0151432. doi: 10.1371/journal.pone.0151432 (PMC4792381; doi:10.1371/journal.pone.0151432)
Supplement: S3 Fig — (PDF) [file pone.0151432.s003.pdf]

S3 Figure. Representative mass spectra of putative flavanone β-triketone conjugates from *E. muelleriana* glands.

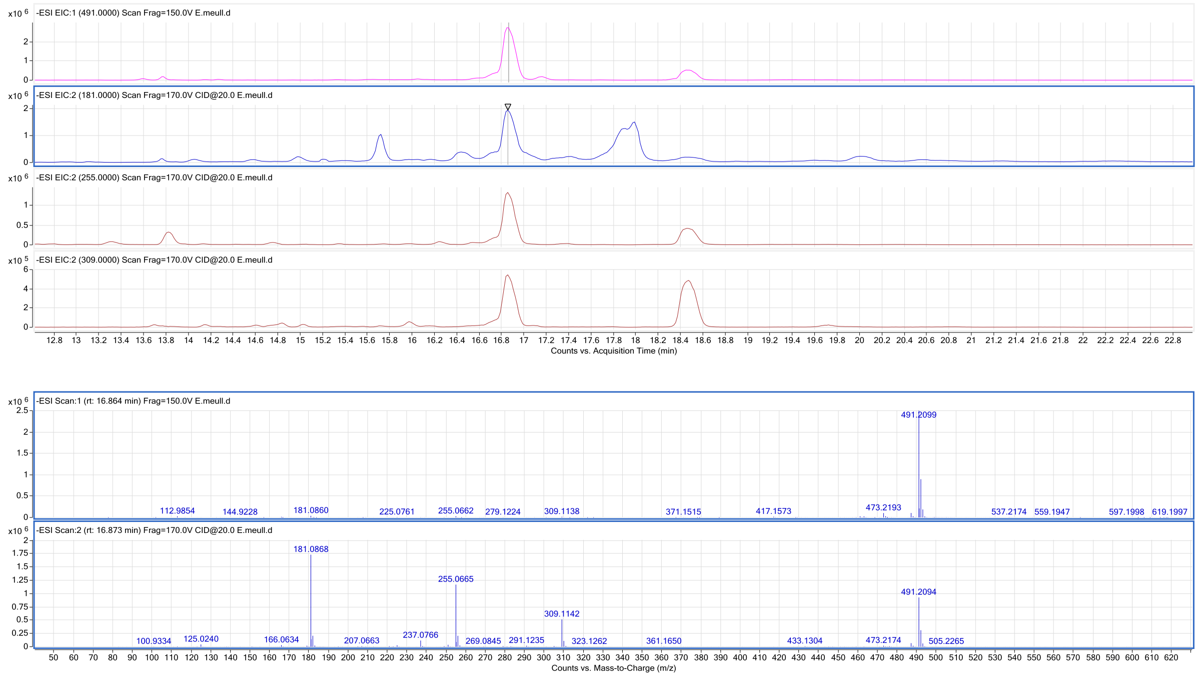

S3A Fig. A putative flavanone β-triketone conjugate from *E. muelleriana* glands observed with *m/z* 491 [M-H]⁻ using ESI-LCMS/MS. Fragmentation creates the ion pair *m/z* 309 and 181 [M-H]⁻ and the flavanone moiety with *m/z* 255. The mass difference between ion 181 and the neutral loss of 236 to create the flavanone is 55 Da, which is characteristic of fragmentation either side of an iso-butyl bridge.

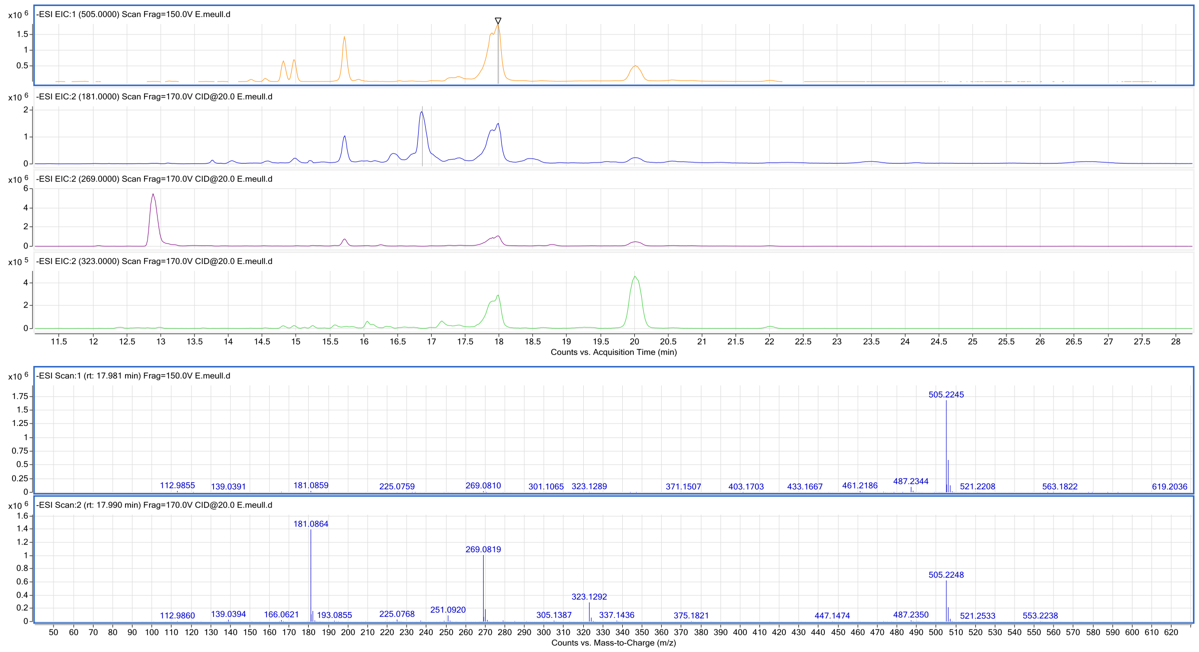

S3B Fig. A second flavanone β-triketone conjugate from *E. muelleriana* glands observed with *m/z* 505 [M-H]⁻. MS2 creates the fragment pair *m/z* 323 and 181 [M-H]⁻, and the flavanone moiety with *m/z*

269. The mass difference between fragment 181 and the neutral loss of 236 (flavanone) is 55 Da indicating fragmentation either side of an iso-butyl bridge.
