# Supplementary material for: Foliar Essential Oil Glands of Eucalyptus Subgenus Eucalyptus (Myrtaceae) Are a Rich Source of Flavonoids and Related Non-Volatile Constituents
Source: PLoS One. 2016 Mar 15;11(3):e0151432. doi: 10.1371/journal.pone.0151432 (PMC4792381; doi:10.1371/journal.pone.0151432)
Supplement: S4 Fig — (PDF) [file pone.0151432.s004.pdf]

S4 Figure. Representative mass spectra of putative β-triketone heterodimers from *E. gregsoniana* glands

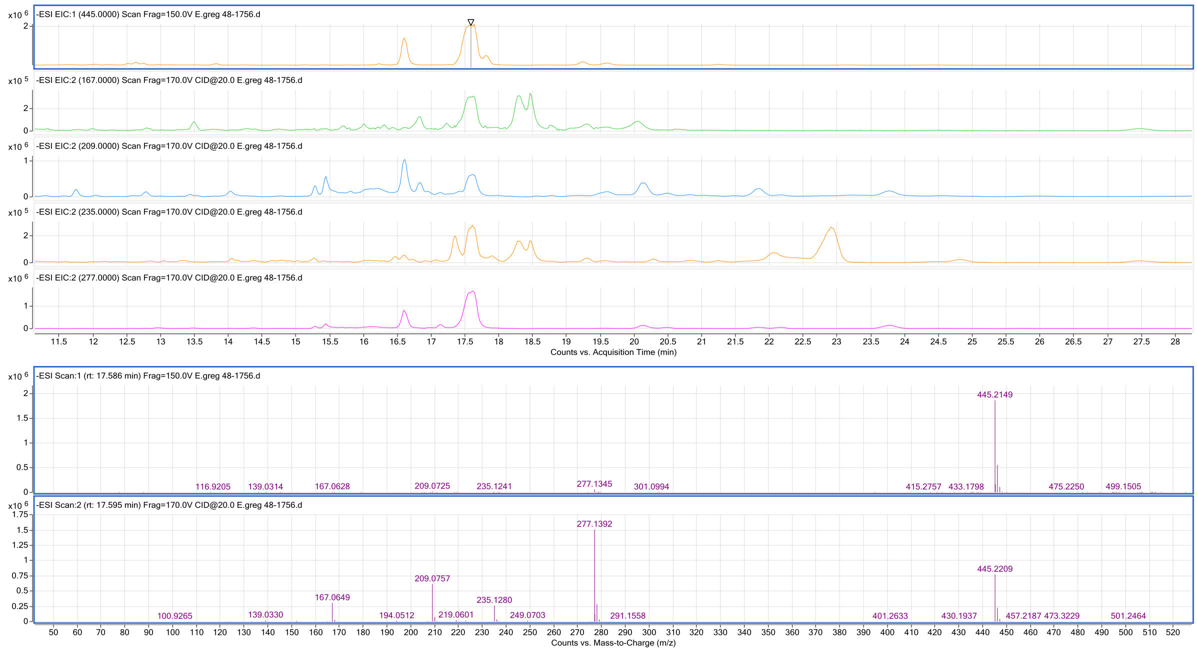

S4A Fig. A putative β-triketone heterodimer from *E. gregsoniana* glands observed with *m/z* 445 [M-H]$^-$ using ESI-LCMS/MS in negative mode. Characteristic fragmentation on either side of the isopentyl bridge creates fragment pairs *m/z* 277 and 167 and *m/z* 235 and 209.

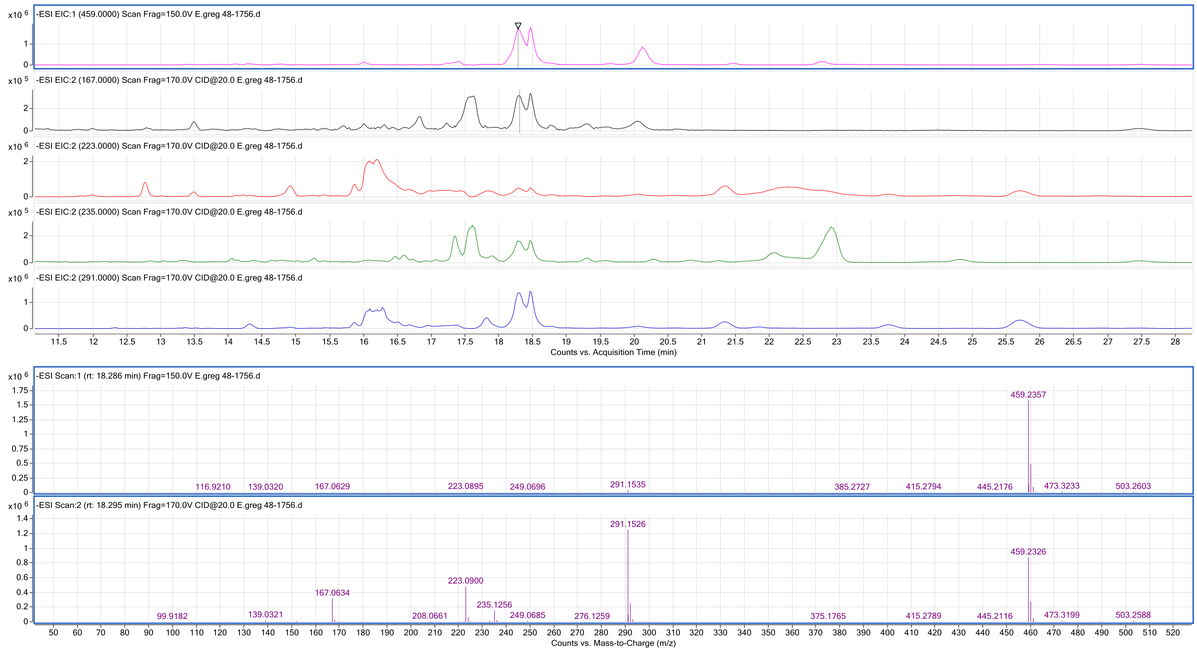

S4B Fig. A second putative β-triketone heterodimer from *E. gregsoniana* glands observed with *m/z* 459 [M-H]$^-$. Characteristic fragmentation on either side of the isopentyl bridge creates fragment pairs *m/z* 291 and 167 and *m/z* 235 and 223.
