# Supplementary material for: Foliar Essential Oil Glands of Eucalyptus Subgenus Eucalyptus (Myrtaceae) Are a Rich Source of Flavonoids and Related Non-Volatile Constituents
Source: PLoS One. 2016 Mar 15;11(3):e0151432. doi: 10.1371/journal.pone.0151432 (PMC4792381; doi:10.1371/journal.pone.0151432)
Supplement: S5 Fig — (PDF) [file pone.0151432.s005.pdf]

<u>S5 Figure. Representative mass spectra of a putative chromone *C*-glucoside from *E. gregsoniana* glands</u>

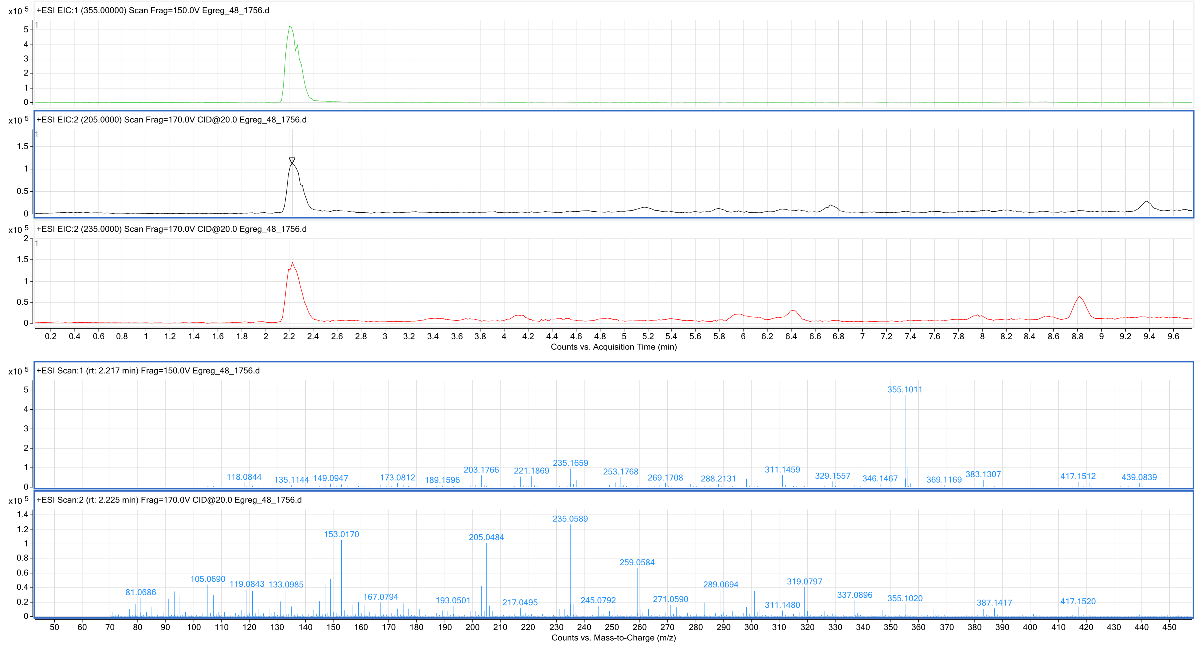

S5 Fig. A putative chromone *C*-glucoside from *E. gregsoniana* glands observed with *m/z* 355 $[M+H]^+$ using ESI-LCMS/MS in positive mode. The characteristic multi-fragment array of *C*-glucosides was observed including an abundant fragment with a neutral loss of 120 Da (*m/z* 235) and relatively less abundant fragment with a loss of 150 Da (*m/z* 205). Similarly characteristic of *C*-glucosides is the very low abundance of the aglycone fragment with *m/z* 193 $[M+H]^+$ exhibiting a neutral loss of 162 Da (glucose).
